# Evaluating the effects of benzoic acid on nursery and finishing pig growth performance

**DOI:** 10.1093/tas/txae049

**Published:** 2024-04-04

**Authors:** Katelyn N Gaffield, Jenna J Bromm, Joel M DeRouchey, Mike D Tokach, Jason C Woodworth, Robert D Goodband, Kiah M Berg, Jon A De Jong, Courtney L Pohlen, Jordan T Gebhardt

**Affiliations:** Department of Animal Sciences and Industry, College of Agriculture, Kansas State University, Manhattan, KS 66506-0201, USA; Department of Animal Sciences and Industry, College of Agriculture, Kansas State University, Manhattan, KS 66506-0201, USA; Department of Animal Sciences and Industry, College of Agriculture, Kansas State University, Manhattan, KS 66506-0201, USA; Department of Animal Sciences and Industry, College of Agriculture, Kansas State University, Manhattan, KS 66506-0201, USA; Department of Animal Sciences and Industry, College of Agriculture, Kansas State University, Manhattan, KS 66506-0201, USA; Department of Animal Sciences and Industry, College of Agriculture, Kansas State University, Manhattan, KS 66506-0201, USA; Pipestone Nutrition, Pipestone, MN56164, USA; Pipestone Nutrition, Pipestone, MN56164, USA; Pipestone Nutrition, Pipestone, MN56164, USA; Department of Diagnostic Medicine/Pathobiology, College of Veterinary Medicine, Kansas State University, Manhattan, KS 66506-0201, USA

**Keywords:** acidifier, benzoic acid, feed additive, finishing pig, growth performance, nursery pig

## Abstract

Three studies were conducted evaluating the use of benzoic acid in swine diets. In experiment 1, 350 weanling barrows (DNA 200 × 400; initially 5.9 ± 0.04 kg) were allotted to one of the five dietary treatments with 14 pens per treatment. Diets were fed in three phases: phase 1 from weaning to day 10, phase 2 from days 10 to 18, and phase 3 from days 18 to 38. Treatment 1 contained no benzoic acid throughout all three phases (weaning to day 42). Treatment 2 included 0.50% benzoic acid throughout all three phases. Treatment 3 contained 0.50% benzoic acid in phases 1 and 2, and 0.25% benzoic acid in phase 3. Treatment 4 contained 0.50% benzoic acid in phases 1 and 2, and no benzoic acid in phase 3. Treatment 5 contained 0.50% benzoic acid in phase 1, 0.25% benzoic acid in phase 2, and no benzoic acid in phase 3. For the overall period, pigs fed 0.50% in the first two phases and 0.25% benzoic acid in the final phase had greater (*P* < 0.05) average daily gain (average daily gain) than pigs fed no benzoic acid through all three phases, or pigs fed 0.50% in the first two phases and no benzoic acid in the final phase, with pigs fed the other treatments intermediate. Pigs fed 0.50% in the first two phases and 0.25% benzoic acid in the final phase had improved (*P* < 0.05) gain-to-feed ratio (G:F) compared with pigs fed no benzoic acid throughout all three phases, pigs fed 0.50% in the first two phases and no benzoic acid in the third phase, or pigs fed 0.50%, 0.25%, and no benzoic acid, respectively. For experiment 2, a 101-d trial was conducted using two groups of 1,053 finishing pigs (2,106 total pigs; PIC 337 × 1,050; initially 33.3 ± 1.9 kg). Dietary treatments were corn–soybean meal-dried distillers grains with solubles-based with the addition of none, 0.25%, or 0.50% benzoic acid. Overall, pigs fed increasing benzoic acid had a tendency for increased average daily feed intake (linear, *P* = 0.083) but decreased G:F (linear, *P* < 0.05). In experiment 3, 2,162 finishing pigs (DNA 600 × PIC 1050; initially 31.4 ± 2.2 kg) were used in a 109-d trial. Dietary treatments were formulated with or without 0.25% benzoic acid. For the overall experimental period, pigs fed benzoic acid had increased (*P* < 0.05) G:F. In summary, feeding benzoic acid elicits improved growth performance when fed throughout the entire nursery period while improved G:F in growing-finishing pigs was observed in one experiment, but not in the other.

## Teaser

Feeding benzoic acid postweaning improves growth performance. However, when subsequently fed a diet without benzoic acid, pigs experienced a reduction in performance. Benzoic acid inclusion in finishing pig diets resulted in inconsistent effects on feed efficiency.

## Introduction

As the swine industry continues to reduce the use of feed-grade antimicrobial growth promoters and pharmacological levels of Zn, finding new alternatives that elicit similar responses has become a focal point (Kongsted and McLoughlin, 2023). A specific class of feed additives that has shown positive effects on growth performance and gut health is organic acids (Suiryanrayna and Ramana, 2015). Acidifiers, such as benzoic acid, are suggested to lower the pH of the gastrointestinal tract leading to potential improvements in nutrient digestion, growth performance, and gut microbiota (Torrallardona et al., 2007; Rao et al., 2023). Furthermore, benzoic acid has been shown to exhibit antimicrobial properties within the gastrointestinal tract (Knarreborg et al., 2002; Outlaw et al., 2023). This mechanism is largely driven by the acidic environment inhibiting the growth of pathogens through the accumulation of hydrogen ions. The acids themselves can also disrupt bacteria cell walls (Nguyen et al., 2020).

Due to these benefits, the effects of benzoic acid on growth performance and intestinal morphology have been widely studied (Torrallardona, et al., 2007; Zhai et al., 2017; Warner et al., 2023). Most studies have reported positive responses in growth performance, but only evaluate one level of the acidifier, often in combination with other feeding strategies (Torrallardona et al., 2007; Zhai et al., 2020; Warner et al., 2023). The few studies that have evaluated multiple benzoic acid feeding levels in nursery diets observed positive responses up to the highest inclusion level (Zhai et al., 2017; Silveria et al., 2018). Despite these findings, previous studies have failed to evaluate the feeding duration throughout the nursery period, thus disregarding an important economical component. Therefore, additional research is needed to further validate benzoic acid feeding strategies throughout the entire nursery period.

Despite the information on benzoic acid inclusion in nursery diets, there has been limited research focusing on the addition of benzoic acid in finishing diets. Recently, a review by Rao et al. (2023) focused on the effects of different feed additives on finishing pig growth performance concluding that acidifiers had the potential to positively impact feed efficiency when compared to other feed additives. However, the challenge with the current research utilizing benzoic acid in finishing diets is the consistency of response. In the feed additive review, implementing acidifiers in finishing diets had an impact on average daily gain (ADG) ranging from −14.9% to 11.4% and an impact on feed efficiency ranging from −9.7% to 11.3% (Rao et al., 2023). More research is needed to understand the consistency in response from feeding finishing pigs diets containing acidifiers. Consequently, the objective of these studies was to evaluate different benzoic acid feeding strategies on nursery pig performance and investigate the effects of benzoic acid supplementation in finishing pig diets.

## Materials and Methods

The Kansas State University Institutional Animal Care and Use Committee approved the protocols used in these experiments (IACUC #4506, #4375, and #4564). Experiment 1 was conducted at the Kansas State University Segregated Early Weaning Facility located in Manhattan, KS. The pigs were housed in two identical barns. Each barn was enclosed and environmentally controlled using mechanical ventilation. Pens had a metal tri-bar floor, contained a cup waterer, and had a four-hole, dry self-feeder. Pens (1.2 × 1.2 m) housed 5 pigs which allowed approximately 0.30 m^2^/pig. Experiment 2 was conducted in two barns at a commercial research grow-finish site located in southwest Minnesota (New Horizon Farms, Pipestone, MN). Each barn had slatted concrete floors, deep pit manure storage, were naturally ventilated, and double-curtain-sided. Pens (3.0 × 5.5 m) contained a cup waterer and a five-hole stainless steel dry self-feeder (Thorp Equipment, Thorp, WI). Pigs were allowed approximately 0.6 m^2^/pig. Experiment 3 was conducted at a commercial research facility located in southwest Minnesota (Pipestone Nutrition, Edgerton, MN). Pigs were housed in a temperature-controlled wean-to-finish facility. Each pen (6.8 × 2.5 m) contained one nipple waterer and a four-hole dry self-feeder. Pigs were allowed approximately 0.6 m^2^/pig. Daily feed additions were recorded for experiments 2 and 3 with a computerized feeding system (FeedPro; Feedlogic Corp., Willmar, MN). Pigs were provided ad libitum access to the treatment diets and water throughout all three experiments.

### Animals and Diets

For experiment 1, a total of 350 weanling barrows (DNA 200 × 400; initially 5.9 ± 0.04 kg) were randomly assigned to pens and pens were allotted to one of the five dietary treatments with 14 replications per treatment. Diets were fed in three phases: phase 1 from weaning to day 10, phase 2 from days 10 to 18, and phase 3 from days 18 to 38. Dietary treatments were formulated to provide 0%, 0.25%, or 0.50% benzoic acid (minimum 99.5% pure benzoic acid; VevoVitall, DSM Nutritional Products, Parsippany, NJ) added at the expense of corn (Table 1). These inclusions were selected as they represent common industry levels fed throughout the nursery period. Treatment 1 served as the control and contained no benzoic acid throughout all three phases. Treatment 2 included 0.50% benzoic acid throughout all three phases. Treatment 3 contained 0.50% benzoic acid for phases 1 and 2, then 0.25% benzoic acid in phase 3. Treatment 4 contained 0.50% benzoic acid in phases 1 and 2, and no benzoic acid in phase 3. Treatment 5 contained 0.50% benzoic acid in phase 1, 0.25% benzoic acid in phase 2, and no benzoic acid in phase 3. For both the phases 1 and 2 diets, a single batch of a base diet was manufactured (Hubbard Feeds, Beloit, KS), then benzoic acid additions were mixed in at the Kansas State University O.H. Kruse Feed Technology Innovation Center, Manhattan, KS. For phase 3, complete diets were manufactured (Hubbard Feeds, Beloit, KS). The diets were fed in meal form and pig weights and feed disappearance were measured on days 0, 10, 18, 24, and 38 to determine ADG, average daily feed intake (ADFI), and gain-to-feed ratio (G:F). Feces were collected on days 10 and 24 from 3 pigs per pen to determine fecal dry matter (DM). Samples were dried at 55 °C for 48 h and loss of weight was used to determine the percentage of fecal DM.

**Table 1.** Diet composition (as-fed basis), experiment 1^1^

Item, %	Phase 1	Phase 2	Phase 3
Ingredient, %			
Corn	44.90	50.15	64.25
Soybean meal	17.40	23.75	31.80
Spray-dried whey	10.00	—	—
Whey permeate	10.00	10.00	—
Dried distillers grains with solubles	5.00	7.50	—
Fish meal	2.50	—	—
Fermented soybean meal^2^	4.00	3.85	—
Spray-dried bovine plasma	2.00	—	—
Choice white grease	1.00	1.00	—
Monocalcium P	0.80	1.00	1.00
Calcium carbonate	0.45	0.45	0.90
Zinc oxide	0.40	0.25	—
Sodium chloride	0.30	0.50	0.60
Vitamin premix with phytase^3^	0.25	0.25	0.25
Trace mineral premix^4^	0.15	0.15	0.15
L-Lys-HCL	0.40	0.55	0.50
DL-Met	0.19	0.22	0.21
L-Thr	0.17	0.22	0.21
L-Trp	0.02	0.04	0.04
L-Val	0.08	0.12	0.13
Benzoic acid^5^	+/−	+/−	+/−
Total	100	100	100
*Calculated analysis*			
Standardized ileal digestible (SID) amino acids, %			
Lys, %	1.35	1.35	1.35
Ile:Lys	57	57	56
Leu:Lys	120	118	115
Met:Lys	36	37	36
Met and Cys:Lys	59	58	58
Thr:Lys	64	63	63
Trp:Lys	19.2	1`9.3	19.4
Val:Lys	70	70	70
Total Lys, %	1.51	1.51	1.50
ME, kcal/kg	3,401	3,373	3,278
NE, kcal/kg	2,542	2,502	2,418
SID Lys:ME, g/Mcal	3.97	4.00	4.12
SID Lys:NE, g/Mcal	5.31	5.40	5.58
Crude protein, %	21.5	21.5	21.3
Ca, %	0.66	0.54	0.71
P, %	0.66	0.61	0.61
STTD P, %	0.58	0.51	0.47

^1^Phase 1 diets were fed from 5.9 to 7.4 kg, phase 2 diets were fed from 7.4 to 10.5 kg, and phase 3 diets were fed from 10.5 to 21.0 kg.

^2^MEPro, Prairie Aquatech, Brookings, SD.

^3^Provided per kg of diet: 4,134 IU vitamin A; 1,653 IU vitamin D; 44 IU vitamin E; 3 mg vitamin K; 0.03 mg vitamin B12; 50 mg niacin; 28 mg pantothenic acid; 8 mg riboflavin. Ronozyme HiPhos GT, DSM Nutritional Products, Parsippany, NJ was included at 1,250 FTU/kg to provide an estimated release of 0.14% STTD P for all diets.

^4^Provided per kg of diet: 110 mg Zn from zinc sulfate; 110 mg Fe from iron sulfate; 33 mg Mn from manganese oxide; 17 mg Cu from copper sulfate; 0.30 mg I from calcium iodate; 0.30 mg Se from sodium selenite.

^5^Benzoic acid (VevoVitall, DSM Nutritional Products, Parsippany, NJ) at none, 0.25%, or 0.50% of the diet was included at the expense of corn.

STTD P, standardized total tract digestible phosphorus.

In experiment 2, a 101-d growth trial was conducted using two groups of 1,053 finishing pigs (2,106 total pigs; PIC 337 × 1,050; initially 33.3 ± 1.91 kg). Pens of pigs (27 pigs per pen), with a similar number of barrows and gilts per pen, were randomly assigned to one of the three dietary treatments in a completely randomized design with 13 replications per treatment in each barn for a total of 26 replications. Dietary treatments were corn–soybean meal-dried distillers grains with solubles-based with the addition of none, 0.25%, or 0.50% benzoic acid. Diets were fed in four phases from 34 to 50, 50 to 75, 75 to 100, and 100 to 132 kg body weight (BW). All treatments were formulated to meet or exceed NRC (2012) requirements for finishing pigs for their appropriate weight ranges (Table 2). All diets were manufactured at New Horizon Farms Feed Mill (Pipestone, MN). Every 2 wk, pens of pigs were weighed and feed disappearance was measured to determine ADG, ADFI, and G:F.

**Table 2.** Diet composition (as-fed basis), experiment 2^1^

Item	Phase 1	Phase 2	Phase 3	Phase 4
Ingredients, %				
Corn	61.09	67.61	71.90	82.91
Soybean meal	26.63	20.40	16.29	15.50
Dried distillers grains with solubles	10.00	10.00	10.00	—
Limestone	1.08	1.00	1.00	0.80
Monocalcium P	0.40	0.25	0.10	0.10
Sodium chloride	0.35	0.35	0.35	0.35
L-Lys-HCl	0.28	0.24	0.22	0.18
DL-Met	0.01	—	—	—
Thr^2^	0.04	0.03	0.01	0.04
Vitamin-trace mineral premix^3^	0.10	0.10	0.10	0.10
Phytase^4^	0.03	0.03	0.03	0.03
Benzoic acid^5^	+/−	+/−	+/−	+/−
Total	100	100	100	100
*Calculated analysis*				
Standardized ileal digestible (SID) amino acids, %				
Lys	1.08	0.90	0.78	0.70
Ile:Lys	67	69	71	69
Leu:Lys	151	165	178	169
Met:Lys	28	30	32	31
Met and Cys:Lys	55	59	63	62
Thr:Lys	61	62	63	65
Trp:Lys	19	19	19	19
Val:Lys	75	79	83	80
His:Lys	45	47	50	49
Total Lys, %	1.24	1.04	0.91	0.80
ME, kcal/kg	3,261	3,261	3,274	3,336
NE, kcal/kg	2,438	2,480	2,506	2,546
SID Lys:ME, g/Mcal	3.31	2.76	2.38	2.10
SID Lys:NE, g/Mcal	4.43	3.63	3.11	2.75
Crude protein, %	20.9	18.4	16.7	14.4
Ca, %	0.56	0.49	0.45	0.37
STTD P, %	0.41	0.36	0.32	0.28

^1^Phases 1, 2, 3, and 4 were fed from approximately 33 to 49.9 kg, 49.9 to 74.8 kg, 74.8 to 99.8 kg, and 99.8 kg to market, respectively.

^2^Thr Pro; CJ America Bio, Downers Grove, IL.

^3^The vitamin and trace mineral premix provided per kg of diet: 5,292 IU vitamin A; 1,323 IU vitamin D; 26.5 IU vitamin E; 2.7 mg vitamin K; 0.02 mg vitamin B12; 49.6 mg niacin; 16.5 mg pantothenic acid; 5.0 mg riboflavin; 16.5 mg Cu; 0.3 mg I; 110.0 mg Fe; 33.1 mg Mn; 0.3 mg Se; and 110.0 mg Zn.

^4^Optiphos Plus (Huevepharma, Sofia, Bulgaria) was included at 750 FTU/kg to provide an estimated release of 0.13% STTD P for all diets.

^5^Benzoic acid (VevoVitall, DSM Nutritional Products, Parsippany, NJ) at none, 0.25%, and 0.50% of the diet was included at the expense of corn.

STTD P, standardized total tract digestible phosphorus.

The three heaviest pigs per pen were visually determined, weighed, and marketed 2 wk prior to the end of the experiment. These pigs were included in growth performance data but were not evaluated for carcass characteristics. At the completion of the experiment, the remaining pens of pigs were weighed and marketed. Pigs were transported to a U.S. Department of Agriculture-inspected packing plant (JSB Swift, Worthington, MN). Carcass data were collected including hot carcass weight (HCW), loin depth, and backfat. Percentage lean was calculated using a proprietary equation from the plant. Carcass yield was calculated using the pen average HCW divided by the pen average final live weight.

In experiment 3, a total of 2,162 finishing pigs (DNA 600 × PIC 1,050; initially 31.4 ± 0.47 kg) were used in a 109-d trial. Dietary treatments were formulated in 2 × 2 factorial with main effects of soybean meal source and benzoic acid. The main effect of soybean meal source was included to accomplish a separate objective and will not be discussed further as there were no interactions with benzoic acid inclusion. Dietary treatments for the main effect of benzoic acid were formulated with or without 0.25% benzoic acid. On day 0, pens were blocked by location in the barn and randomly allotted to one of the two benzoic acid treatment levels. There were 27 or 28 pigs per pen and 40 pens per benzoic acid treatment. A similar number of barrows and gilts were placed in each pen. Experimental diets were fed in six different phases. Pigs were fed by a feed budget with phases 1, 2, 3, 4, and 5 provided at 19, 42, 49, 50, and 45 kg per pig, respectively. Phase 6 was provided for the remainder of the study until pigs were marketed. Pens of pigs and feed disappearance were measured approximately every 14 d to determine ADG, ADFI, and G:F. All nutrients were formulated to meet or exceed NRC (2012) requirement estimates (Table 3). All diets were corn–soybean meal-based and were fed in meal form. Diets were manufactured at the Spronk Brothers Feed Mill (Edgerton, MN).

**Table 3.** Diet composition (as-fed basis), experiment 3^1^

	Phase 1		Phase 2		Phase 3		Phase 4		Phase 5		Phase 6	
Ingredient, %	SBM	EESBM	SBM	EESBM	SBM	EESBM	SBM	EESBM	SBM	EESBM	SBM	EESBM
Corn	64.55	60.04	68.95	65.56	73.98	71.70	77.33	75.66	78.24	76.34	85.72	84.27
Soybean meal	22.60	—	18.35	—	—	—	—	—	—	—	12.35	—
EESBM^2^	—	27.04	—	21.67	—	—	—	—	—	—	—	13.75
DDGS	10.00	10.00	10.00	10.00	10.00	10.00	10.00	10.00	10.00	10.00	—	—
Limestone	0.88	0.88	0.88	0.88	0.85	0.85	0.83	0.83	0.78	0.78	0.68	0.68
Monocalcium phosphate	0.34	0.35	0.32	0.33	0.28	0.28	0.22	0.23	0.13	0.13	0.32	0.33
Sodium chloride	0.50	0.50	0.50	0.50	0.50	0.50	0.50	0.50	0.50	0.50	0.50	0.50
L-Lys-HCl	0.55	0.58	0.51	0.54	0.48	0.50	0.46	0.47	0.40	0.40	0.24	0.26
DL-Met	0.16	0.19	0.12	0.14	0.08	0.09	0.05	0.06	0.02	0.03	0.00	0.00
L-Trp	0.03	0.03	0.03	0.03	0.03	0.03	0.03	0.03	0.02	0.02	—	0.01
Thr^3^	0.24	0.24	0.21	0.21	0.19	0.19	0.16	0.16	0.14	0.13	0.08	0.08
L-Val	0.04	0.03	0.02	0.02	—	—	—	—	—	—	—	—
Vitamin and trace mineral premix	0.10	0.10	0.10	0.10	0.10	0.10	0.10	0.10	0.10	0.10	0.10	0.10
Copper chloride	0.03	0.03	0.03	0.03	0.03	0.03	0.03	0.03	0.03	0.03	0.03	0.03
Phytase^4^	0.01	0.01	0.01	0.01	0.01	0.01	0.01	0.01	0.01	0.01	0.01	0.01
Benzoic acid^5^	+/−	+/−	+/−	+/−	+/−	+/−	+/−	+/−	+/−	+/−	+/−	+/−
Total	100	100	100	100	100	100	100	100	100	100	100	100
*Calculated analysis*												
Standardized ileal digestible (SID) amino acids, %												
Lys	1.20	1.27	1.07	1.12	0.93	0.95	0.83	0.85	0.77	0.79	0.67	0.69
Ile:Lys	55	55	55	55	55	55	55	55	58	58	64	63
Leu:Lys	128	123	134	130	143	139	151	148	160	158	166	160
Met and Cys:Lys	58	58	58	58	58	58	58	58	58	58	60	58
Thr:Lys	63	63	63	63	64	64	64	64	65	65	66	66
Trp:Lys	17	17	17	17	17	17	17	17	17	17	17	17
Val:Lys	65	65	65	65	65	65	66	66	70	70	76	75
ME, kcal/kg	3,186	3,366	3,208	3,353	3,232	3,339	3,252	3,333	3,258	3,336	3,280	3,375
NE, kcal/kg	2,458	2,542	2,480	2,548	2,506	2,557	2,524	2,562	2,531	2,567	2,545	2,592
SID Lys:ME, g/Mcal	3.76	3.76	3.33	3.33	2.85	2.85	2.54	2.54	2.01	2.01	2.00	2.01
SID Lys:NE, g/Mcal	4.88	5.00	4.31	4.40	3.71	3.72	3.29	3.32	3.04	3.07	2.63	2.66
Crude protein, %	19.02	19.57	17.34	17.76	15.42	15.66	14.16	14.31	13.90	14.17	13.01	13.00
Ca, %	0.60	0.60	0.58	0.58	0.55	0.55	0.52	0.52	0.48	0.48	0.48	0.48
STTD P, %	0.37	0.37	0.36	0.36	0.34	0.34	0.32	0.32	0.30	0.30	0.31	0.31

^1^Pigs were fed on a feed budget with phase 1, 2, 3, 4, and 5 provided at 19, 42, 49, 50, and 45 kg per pig, respectively. Phase 6 was provided for the remainder of the study.

^2^Lester Feed and Grain (Lester, IA).

^3^Thr Pro; CJ America Bio, Downers Grove, IL.

^4^Quantum Blue 5P (AB Vista, Marlborough, UK) was included at 2,000 FTU/kg to provide an estimated release of 0.11% STTD P for all diets.

^5^VevoVitall (DSM Products, Parsippany, NJ) was added at 0.25%.

SBM, soybean meal; EESBM, extruded-expelled soybean meal; DDGS, dried distillers grains with solubles.

On days 88 and 96, eight of the heaviest pigs per pen were visually selected, weighed individually, and transported to a commercial packing plant (WholeStone Farms, Fremont, NE) for processing and carcass data collection. The remaining pigs were marketed at the conclusion of the trial on day 109 and transported to WholeStone Farms for carcass data collection. A fat sample was taken from the belly of one barrow per pen per marketing event and included all three layers of fat. Analysis of iodine value was conducted using near-infrared spectroscopy at WholeStone Farms.

### Statistical Analysis

For experiment 1, data were analyzed as a completely randomized design with pen serving as the experimental unit, treatment as a fixed effect, and barn as a random effect. Data were analyzed using R Studio (Version 3.5.2, R Core Team, Vienna, Austria). Contrasts were used to test for the main effects of the different benzoic acid feeding levels (0%, 0.25%, and 0.50%), within the three phases. Overall performance data were analyzed as a one-way ANOVA using the lmer function from the lme4 package. Differences for treatments demonstrating a significant source of variation were determined through pairwise comparisons using the Tukey-Kramer multiplicity adjustment to control for type I error. Similarly, contrasts were used to test for the main effects of treatment, day, and interaction between treatment and day of different benzoic feeding levels on fecal DM.

For experiment 2, data were analyzed as a completely randomized design with pen as the experimental unit and treatment as the fixed effect. Data were analyzed as a one-way ANOVA using the lmer function from the lme4 package in R (version 4.1.1 [August 10, 2021], R Foundation for Statistical Computing, Vienna, Austria). Contrasts were used to test the main effect of benzoic acid levels (0%, 0.25%, and 0.50%). Similarly, contrasts were used to analyze carcass characteristics including backfat, loin depth, and percentage lean with HCW serving as a covariate.

For experiment 3, data were analyzed using the GLIMMIX procedure of SAS OnDemand for Academics (SAS Institute, Inc., Cary, NC) in a randomized complete block design with pen as the experimental unit and location as the blocking factor. Treatments were considered a fixed effect and block as a random effect. The main effect of benzoic acid (0% vs. 0.25%) was analyzed. For all three experiments, results were considered significant at *P* ≤ 0.05 and marginally significant at 0.05 > *P* ≤ 0.10.

## Results

### Experiment 1

From days 0 to 10 (phase 1), pigs fed 0.50% benzoic acid had increased (*P* ≤ 0.05) ADG, G:F, and heavier day 10 BW than those fed the control diet (Table 4). From days 10 to 18 (phase 2), pigs fed 0.50% benzoic acid had increased (*P* < 0.01) ADG compared to pigs fed either none or 0.25% benzoic acid, while pigs fed 0.25% benzoic acid had decreased (*P* < 0.001) G:F compared to pigs fed none or 0.50% benzoic acid. There was a significant increase in ADFI for pigs fed 0.25% (*P *= 0.033) benzoic acid and a marginally significant increase in ADFI for pigs fed 0.50% (*P *= 0.069) benzoic acid in phase 2 compared to pigs fed no benzoic acid; however, they did not differ from each other. Pigs fed 0.50% benzoic acid had increased (*P* = 0.012) day 18 BW compared to pigs fed no benzoic acid, while pigs fed 0.25% benzoic acid were intermediate. From days 0 to 18 (phases 1 and 2), pigs fed 0.50% benzoic acid had an increased (*P* = 0.013) ADG compared to pigs fed no benzoic acid, but there was no evidence of differences (*P* > 0.10) in ADFI or G:F. When looking at the individual feeding programs for the combined phases 1 and 2 period, there were no differences (*P* > 0.10) in ADG or ADFI. However, pigs fed 0.50% benzoic acid in phase 1 and 0.25% benzoic acid in phase 2 had a lower (*P* < 0.05) G:F than pigs fed 0.50% benzoic acid throughout both phases, with pigs fed no benzoic acid being intermediate. From days 18 to 38 (phase 3), pigs fed 0.50% or 0.25% benzoic acid had increased (*P* < 0.01) ADG and ADFI compared with pigs fed no benzoic acid. Additionally, pigs fed 0.25% benzoic acid in phase 3 had improved (*P *< 0.05) G:F compared to pigs fed none or 0.50% benzoic acid.

**Table 4.** Effects of benzoic acid feeding strategy on nursery pig performance, experiment 1^1^^,^^2^

	Benzoic acid, %^3^								
Phase 1	0	0.50	0.50	0.50	0.50				
Phase 2	0	0.50	0.50	0.50	0.25		Contrast, *P* =		
Phase 3	0	0.50	0.25	0	0	SEM	0% vs 0.50%	0% vs 0.25%	0.25% vs 0.50%
BW, kg									
day 0	5.9	5.9	5.9	5.9	5.9	0.04	0.785	—	—
d 10	7.3	7.5	7.4	7.4	7.6	0.09	0.040	—	—
d 18	10.2	10.7	10.6	10.5	10.4	0.14	0.012	0.186	0.340
d 38	20.4^bc^	21.6^ab^	21.9^a^	20.2^c^	20.8^abc^	0.36	0.003	<0.001	0.553
d 0 to 10 (Phase 1)									
ADG, g	136	164	147	150	172	10.0	0.034	—	—
ADFI, g	164	185	176	171	193	10.5	0.144	—	—
G:F, g/kg	821	891	839	878	896	27.3	0.045	—	—
d 10 to 18 (Phase 2)									
ADG, g	363	395	401	382	350	10.0	0.009	0.333	<0.001
ADFI, g	454	485	483	472	492	12.5	0.069	0.033	0.412
G:F, g/kg	806	816	832	811	712	20.0	0.483	<0.001	<0.001
d 0 to 18 (Phase 1 and 2)^4^									
ADG, g	236	265	257	252	251	7.4	0.013	—	—
ADFI, g	292	317	309	303	326	10.0	0.141	—	—
G:F, g/kg	813^ab^	840^a^	832^a^	834^a^	771^b^	11.7	0.106	—	—
d 18 to 38 (Phase 3)									
ADG, g	510	544	564	485	515	14.7	0.003	<0.001	0.233
ADFI, g	771	825	821	754	784	24.4	0.004	0.008	0.869
G:F, g/kg	662	660	687	644	658	7.1	0.504	<0.001	0.009
d 0 to 38 (Overall)									
ADG, g	380^bc^	410^ab^	417^a^	374^c^	390^abc^	10.1	—	—	—
ADFI, g	543	581	575	539	567	16.4	—	—	—
G:F, g/kg	700^b^	706^ab^	724^a^	694^b^	689^b^	6.2	—	—	—
Fecal DM, %^5^									
d 10	26.32	26.62	24.65	26.18	26.68	0.675	0.739	—	—
d 18	23.58	24.02	23.54	24.51	24.57	0.675	0.450	0.173	0.356

^1^A total of 350 weanling barrows (DNA 200 × 400; initially 5.9 ± 0.04 kg) approximately 21 d of age were used in a 38-d experiment with 5 pigs per pen and 14 pens per treatment.

^2^Different superscript letters within the same row reflect dietary treatment differences (*P* ≤ 0.05).

^3^VevoVitall, DSM Nutritional Products, Parsippany, NJ.

^4^The 0% vs 0.5% contrast for d 0 to 18 compares only the treatments that had the same inclusions of benzoic acid throughout both phases 1 and 2. A contrast statement is not used to compare the final treatment as there was a reduction in the benzoic acid inclusion between the two phases.

^5^Feces from three piglets from each pen were pooled, weighed, and dried to measure fecal dry matter. Treatment × day, *P* = 0.679; treatment, *P* = 0.199; day, *P* < 0.001.

For the overall experimental period (days 0 to 38), pigs fed 0.50% benzoic acid in the first two phases and 0.25% benzoic acid in the final phase had a greater (*P* < 0.05) ADG than pigs fed no benzoic acid through all three phases and pigs fed 0.50% benzoic acid in the first two phases with no benzoic acid in the final phase, while pigs fed the other treatments were intermediate. Pigs fed 0.50% in phases 1 and 2 and 0.25% benzoic acid in the final phase had increased (*P* < 0.05) G:F compared with pigs fed no benzoic acid throughout all three phases, pigs fed 0.50% in the first two phases and no benzoic acid in the third phase, and pigs fed 0.50%, 0.25%, and no benzoic acid, respectively. There was also evidence for differences (*P *< 0.01) in day 38 BW with pigs fed 0.50% benzoic acid in the first two phases and 0.25% benzoic acid in the third phase having increased (*P *< 0.01) BW compared with pigs fed no benzoic acid throughout all three phases and pigs fed 0.50% benzoic acid in the first two phases with no benzoic acid in the final phase. There was no evidence (*P *> 0.10) of an interaction between treatment and day for fecal DM. Furthermore, there was no evidence of a main effect of treatment (*P *> 0.10) for fecal DM. However, there was evidence for a main effect of day (*P *< 0.001) with greater fecal DM on days 10 vs. 18.

### Experiment 2

In the grower period (days 0 to 44), there was no evidence of differences (*P* > 0.10) for any growth response criteria (Table 5). For the finisher period (days 44 to 101), increasing benzoic acid tended to increase ADFI (linear, *P* = 0.053) but decrease G:F (linear, *P* = 0.002). There was no evidence for differences (*P* > 0.10) in ADG. Similarly, for the overall experimental period (days 0 to 101), pigs fed increasing benzoic acid had a tendency for increased ADFI (linear, *P* = 0.083) and decreased G:F (linear, *P* = 0.011). There was no evidence of difference in ADG (*P* > 0.10) for the overall experimental period. Furthermore, there was no evidence of differences (*P* > 0.10) in grower BW (day 44) or final BW (day 101).

**Table 5.** Effects of increasing benzoic acid on grow-finish pigs growth performance and carcass characteristics, experiment 2^1^

	Benzoic acid, %^2^				*P* =	
Item	0	0.25	0.50	SEM	Linear	Quadratic
BW, kg						
Initial (day 0)	33.3	33.4	33.3	1.91	0.996	0.900
Grower (day 44)	70.3	70.4	71.0	1.79	0.574	0.774
Final (day 101)	125.4	125.7	125.9	1.14	0.707	0.977
Grower (days 0 to 44)						
ADG, kg	0.84	0.84	0.86	0.011	0.389	0.597
ADFI, kg	2.02	2.02	2.07	0.054	0.188	0.588
G:F, g/kg	418	416	414	7.2	0.380	0.983
Finisher (days 44 to 101)						
ADG, kg	0.99	0.99	0.98	0.043	0.616	0.868
ADFI, kg	3.05	3.05	3.11	0.132	0.053	0.340
G:F, g/kg	325	323	317	1.8	0.002	0.303
Overall						
ADG, kg	0.92	0.92	0.93	0.026	0.777	0.630
ADFI, kg	2.59	2.59	2.64	0.085	0.083	0.404
G:F, g/kg	357	355	351	2.2	0.011	0.472
Carcass characteristics						
HCW, kg	92.1	92.5	93.1	0.73	0.254	0.956
Carcass yield, %	72.4	72.5	72.7	0.24	0.359	0.828
Lean, %^3^	56.5	56.4	56.4	0.24	0.745	0.490
Backfat, mm^3^	16.1	16.1	16.0	0.34	0.862	0.591
Loin depth, mm^3^	61.9	61.4	61.3	0.37	0.134	0.487

^1^A total of 2,106 pigs (PIC 337 × 1,050; initially 33.3 ± 1.91 kg) were used in two groups with 27 pigs per pen and 26 replicates per treatment.

^2^VevoVitall, DSM Nutritional Products, Parsippany, NJ.

^3^Adjusted using hot carcass weight as covariate.

For carcass characteristics, no evidence of difference (*P* > 0.10) was observed for any criteria including HCW, carcass yield, backfat, loin depth, or percentage lean due to increasing benzoic acid.

### Experiment 3

From days 0 to 51, pigs fed diets without benzoic acid had greater (*P* ≤ 0.01) ADG and ADFI compared to pigs fed diets containing benzoic acid (Table 6). Pigs fed diets containing benzoic acid had increased (*P* < 0.001) G:F compared to pigs fed diets without benzoic acid. From days 51 to 109, there was a tendency for an increase (*P* = 0.06) in ADG in pigs fed diets containing benzoic acid compared to those fed diets without benzoic acid. There were no effects (*P* > 0.10) of benzoic acid on ADFI or G:F during this period. Overall (days 0 to 109), pigs fed benzoic acid had decreased (*P* = 0.02) ADFI, but similar (*P* > 0.10) ADG. As a result, pigs fed benzoic acid had improved (*P* = 0.01) G:F compared to pigs fed diets without benzoic acid. Carcass characteristics and iodine value were not impacted (*P* > 0.10) by benzoic acid treatment.

**Table 6.** Main effect of benzoic acid on growth performance, carcass characteristics, and carcass iodine value, experiment 3^1^

	Benzoic acid^2^			
Item	None	0.25%	SEM	*P* =
BW, kg				
Initial (day 0)	31.4	31.3	0.47	0.85
Grower (day 51)	78.6	77.2	0.71	0.03
Final (day 109)	123.5	122.8	0.74	0.39
Grower (days 0 to 51)				
ADG, kg	0.92	0.90	0.08	0.01
ADFI, kg	2.03	1.91	0.02	< 0.001
G:F, g/kg	456	470	2.7	< 0.001
Finisher (days 51 to 109)				
ADG, kg	0.92	0.94	0.01	0.06
ADFI, kg	2.87	2.91	0.02	0.14
G:F, g/kg	322	324	1.6	0.38
Overall (days 0 to 109)				
ADG, kg	0.92	0.92	0.01	0.37
ADFI, kg	2.43	2.38	0.02	0.02
G:F, g/kg	380	385	1.5	0.01
Carcass characteristics				
HCW, kg	91.5	90.9	0.67	0.18
Yield, %	73.51	73.47	0.01	0.68
Iodine value	70.25	70.52	0.27	0.43

^1^A total of 2,162 pigs (DNA 600 × PIC 1,050; initially 31.4 ± 0.47 kg) were used with 27 to 28 pigs per pen and 40 replications per benzoic acid treatment for a 109-d trial.

^2^VevoVitall (DSM Products, Parsippany, NJ) was added at 0.25%.

SBM, soybean meal; EESBM, extruded-expelled soybean meal.

## Discussion

As the swine industry decreases the use of antimicrobial growth promoters and pharmacological levels of Zn, researchers have begun to investigate multiple feed additives including acidifiers, prebiotics, probiotics, phytogenics, nucleotides, and direct-fed microbials in order to maintain pig health and growth performance (Liu et al., 2018). The growing interest in acidifiers is largely driven by the proposed multi-mechanistic effects driven by the lowered gastric pH. These effects are outlined by Liu et al. (2018) and include (1) increased pepsin activity, (2) inhibition of pathogenic bacteria, and (3) improved nutrient digestibility.

Benzoic acid is an aromatic carboxylic acid and is classified as a weak organic acid. When added at 0.50% in a complete swine diet, benzoic acid has the ability to reduce the calculated acid-binding capacity-4 by approximately 30 mEq/kg (Warner et al., 2023). Although benzoic acid tends to be less acidic than other organic acids, such as formic acid, it has been shown to successfully decrease the pH of stomach contents when fed at levels ranging from 0.20% to 0.75% (Chen et al., 2017; Silveira et al., 2018). In addition to acidification of the gastrointestinal tract, benzoic acid has been shown to reduce urine pH through excreting the metabolite, hippuric acid.

Weaning is a stressful event in a pig’s life and typically occurs during a critical window of immune and intestinal development (Moeser et al., 2017). During this time, weaned pigs exhibit reduced stomach acid secretions which do not typically peak until 56 d of age (Yen, 2001; Pluske, 2016). Thus, a pig is challenged with a multitude of factors including changes in environment, diet, and physiological development at the time of weaning. Therefore, by the addition of an acidifier in nursery diets, there is a potential for a reduction in pH in the digestive tract. This reduction in pH has the potential to assist a young pig through antimicrobial effects, increased nutrient digestion, and improved intestinal health, ultimately yielding improved growth performance (Liu et al., 2018; Tugnoli et al., 2020). Many studies utilizing benzoic acid in nursery diets only evaluate one level of the acidifier often in combination with other feeding strategies (Guggenbuhl et al., 2007; Torrallardona et al., 2007; Zhai et al., 2020; Warner et al., 2023). These studies all found significant improvements in growth performance including increased ADG and G:F with 0.50% benzoic acid added to the diet. Furthermore, the few studies that have evaluated multiple benzoic acid feeding levels in nursery diets observed positive responses up to the highest levels of inclusion, 0.50% and 0.75% (Zhai et al., 2017; Silveria et al., 2018). Despite previous literature evaluating the growth performance of nursery pigs supplemented with benzoic acid, there is currently no research investigating feeding duration and level throughout the nursery period.

A review by Kil et al. (2011) outlined the results of multiple previous studies utilizing benzoic acid in the nursery period and found a positive response during the first 2 wk postweaning. However, according to experiment 1, it appears that feeding 0.50% benzoic acid in all three phases or 0.50% benzoic acid for the first two phases and 0.25% in the third phase will result in the best growth performance for nursery pigs. The reduction in performance reported when benzoic acid was removed from the diet both when transitioning from phase 1 to 2 and when transitioning from phase 2 to 3 is a unique finding of this study. One potential explanation for this decrease in performance is a change in feed palatability when benzoic acid is removed from the diet. A study by Partanen et al. (2002) reported that pigs preferred diets containing sodium benzoate compared to other organic acids such as formic and lactic acid. However, in experiment 1, there was no decrease in feed intake witnessed when benzoic acid was reduced in the diet in phase 2. Therefore, the reduction in ADG may be attributed to shifts in gastrointestinal pH or digestibility when benzoic acid levels change. Furthermore, since pigs were 18 d postweaning and approximately 10.5 kg BW when this change from phase 2 to phase 3 occurred, this might indicate their gastrointestinal system had not matured enough in physiological development to handle a less complex diet. Ultimately, further research is needed to truly understand the mechanism behind this decrease in growth performance.

Similar to the nursery period, implementing the optimal level and duration of benzoic acid in growing-finishing diets presents unique challenges. A review by Rao et al. (2023) focusing on the effects of feed additives on finishing pig growth performance showed acidifiers had a potential positive impact on feed efficiency when compared to other feed additives. However, the challenge with the current research utilizing benzoic acid in finishing diets is the consistency of response. In the feed additive review, implementing acidifiers in finishing diets had a wide impact on growth performance with effects on ADG ranging from −14.9% to 11.4% and feed efficiency ranging from −9.7% to 11.3% (Rao et al., 2023). The data in experiment 2 investigating the effects of feeding increasing levels of benzoic acid suggests that feeding benzoic acid in the grow-finish period had no effect on ADG, but tended to increase ADFI and decreased G:F by 1.7%. These data contradict past studies showing a positive (Zhai et al., 2017) or no response (Cho et al., 2014; O’Meara et al., 2020) to benzoic acid supplementation in the growing-finishing period. The consistency of response to benzoic acid supplementation has proven to be more variable during the grow-finish production phase compared to the nursery period across past literature. This inconsistency in response was demonstrated further in experiment 3 where G:F improved by 1.1% when 0.25% benzoic acid was added to the diet.

In summary, these data suggest that feeding benzoic acid for the first 38-d postweaning improves ADG and G:F. However, when benzoic acid was removed from the nursery diet, pigs experienced a reduction in performance and ultimately had similar performance to pigs fed no benzoic throughout the entire experimental period. In this study, feeding 0.50% benzoic acid in all three phases or 0.50% benzoic acid for the first two phases and 0.25% in the third phase resulted in the best growth performance throughout the nursery period. Furthermore, the two finisher trials conducted demonstrate the inconsistency of response to benzoic acid supplementation in finishing diets. One trial suggests that feeding benzoic acid in the grow-finish period had no impact on ADG, but tended to increase ADFI and worsen G:F while the other study found that additions of benzoic acid improved feed efficiency. Overall, further research is warranted to better understand under what conditions a positive response might repeatedly be observed in the growing-finishing period as well as understanding the carry-over effect of benzoic acid supplementation from the nursery into the finishing period.
